# Optimization of waste biomass demineralization through response surface methodology and enhancement of thermochemical and fusion properties

**DOI:** 10.1038/s41598-024-63471-4

**Published:** 2024-11-08

**Authors:** Asif Nadeem Tabish, Muhammad Irfan, Muneeb Irshad, Muhammad Asif Hussain, Hassan Zeb, Saad Jahangir, Akmal Shahzad, Muhammad Hamid Siddiqi, M. A. Mujtaba, Yasser Fouad, M. A. Kalam

**Affiliations:** 1 Department of Chemical Engineering, UET Lahore (New Campus), Lahore, 39021 Pakistan; 2 Department of Physics, UET Lahore, Lahore, 54890 Pakistan; 3 Institute of Metallurgy and Materials Engineering, University of the Punjab, Lahore, 54590 Pakistan; 4 Institute of Energy and Environmental Engineering, University of the Punjab, Lahore, 54590 Pakistan; 5 Automotive Engineering Centre, UET Lahore, Lahore, 54890 Pakistan; 6 Center for Energy Research and Development (CERAD), UET Lahore (New Campus), Lahore, 39021 Pakistan; 7 School of Energy and Power Engineering, Xi’an Jiaotong University, Xi’an, China; 8 Department of Mechanical Engineering, UET Lahore (New Campus), Lahore, 54890 Pakistan; 9 Department of Applied Mechanical Engineering, College of Applied Engineering, Muzahimiyah Branch, King Saud University, P.O. Box 800, 11421 Riyadh, Saudi Arabia; 10 School of Civil and Environmental Engineering, FEIT, University of Technology Sydney, Ultimo, NSW 2007 Australia

**Keywords:** Demineralization, Sugarcane bagasse, Response surface methodology, Lignocellulose, TGA, Energy science and technology, Engineering, Materials science

## Abstract

This study examines the impact of leaching with dilute hydrochloric acid solution on the reduction of ash content and the thermal degradation behavior of sugarcane bagasse. Response surface methodology (RSM) was used to statistically design the experiments and investigate the effect of three independent variables: treatment time, solid-to-liquid ratio, and reagent concentration. The leaching conditions were further optimized and experimentally validated for maximum ash reduction for suitability of treated biomass as feedstock for thermochemical conversion technologies. Reagent concentration and treatment time directly affected ash reduction, while the solid-to-liquid ratio inversely influenced it. Concentration had the highest impact, and treatment duration had the least. The maximum 78.2% ash reduction was achieved by treating the biomass with 1 M HCl for 80 min at a solid-to-liquid ratio of 50:1 (wt/vol). This ash reduction also resulted in a 9.82% increase in higher heating value (HHV). Hemicellulose hydrolysis during leaching was observed through chemical composition and Fourier-transform infrared spectroscopy (FTIR). Ash fusion temperatures increased, indicating more thermally stable biomass. Thermogravimetric analysis (TGA) showed elevated maximum degradation temperature and activation energy.

## Introduction

Biomass is a primitive and carbon-based renewable energy source known to humankind. In agrarian countries like Pakistan, short-term rotation crop residues such as cotton straw, wheat straw, sugarcane bagasse, rice husk, and maize stalks are produced in huge volumes annually. Pakistan is the sixth-largest cane sugar producer and the fifth-largest sugar consumer^1^. The cultivation of sugarcane covers approximately 1.2 million hectares of land, accounting for around 4.5% of the irrigated land in the country. As a byproduct of cane sugar production, sugarcane bagasse is obtained in a huge volume and conventionally burned in the boilers to generate steam and power alike other agricultural residues. When burnt directly and inefficiently, it reduces the resource's value and increases pollution levels and the risk of respiratory illnesses like asthma, bronchitis, emphysema, and even cancer^2^. This issue can be significantly mitigated by repurposing agricultural residues for energy and biofuel production^3^. Therefore, the sustainable exploitation of agricultural wastes is an important subject of sustainable development, and scientists, engineers, policymakers, and governments are engaged in this field.

There is a significant interest in the biomass valorization and thermochemical conversion of agricultural residues to produce biofuels, in addition to the generation of heat and power^4–7^. The agricultural residue consists of lignin, cellulose, hemicellulose, and a considerable fraction of ash forming inorganic components. Depending on its type and origin, the inorganic component can account for up to 18.4 wt% of the total biomass, while the acceptable ash concentration for thermochemical conversion technologies such as combustion, gasification, and pyrolysis is less than 1 wt%^8–10^. During the thermochemical conversion of biomass, it is important to pay special attention to alkali and alkaline earth metals (AAEMs) present in the ash. These metals can serve as catalysts for the degradation of biomass and can also react with SiO_2_ and Al_2_O_3_ to produce silicate and other low melting eutectoids^11^. The high flame temperature that occurs during combustion causes these eutectoids to melt and adhere to the surfaces of the boiler, generating a strong and fused glassy layer^12^ that prevents heat transfer and causes surface corrosion, thus lowering the system's overall thermal efficiency. During the pyrolysis process for bio-oil production, AAEMs serve as catalysts and have significant potential to decrease the bio-oil yield and its stability^13^. The ash-related side reactions not only lead to slagging, agglomeration, corrosion, heat-exchanger fouling, and poor ash fusion characteristics^14–16^ but also result in various technical challenges and threats to the environment and the health of those affected^17^. These are conceivably crucial design and operation restrictions for thermochemical conversion technologies^18^. Therefore, changes in biomass feedstocks' composition are unavoidable to lessen the amount of inorganics present before thermochemical conversions.

Pre-treatment techniques such as water-washing and chemical leaching have been suggested to demineralize the ash-forming components from biomass feed stocks^19^. Water washing is an effective, quick, and inexpensive technique to substantially reduce the inorganic fractions in biomass^15,20,21^. Water washing is a viable method for removing water-soluble alkali compounds such phosphates, nitrates, and chlorides. However, it is not efficient in removing cations that are linked to reactive sites^18,22^. Acid leaching is superior to water washing for ash reduction because acids are equally effective at removing cations linked to reactive sites and water-soluble minerals^23^. Moreover, potassium, sodium, ammonium, and calcium hydroxides have been proven to be efficient in eliminating silica, alumina, and various sulfur-based compounds^19^. The amorphous structure of biomass can also react with the acidic and basic reagents, facilitating the hydrolysis and delignification of biomass^2,24^. As a result, the energy content is decreased, and the biomass resource is valued less when organic parts are removed. Although reagent nature plays a significant role in determining the energy content, other factors such as concentration, temperature, treatment time, and solution pH can also significantly impact treatment effectiveness and energy content. Thus, the treatment of biomass feedstock requires a clear understanding of the appropriate reagent and treatment conditions.

Demineralization of sugarcane bagasse by chemical pretreatment has been a subject of numerous studies utilizing deionized water^25^, sulfuric acid^26^, hydrochloric acid^27^, hydrofluoric acid^5^, nitric acid^26,28^, phosphoric acid^29^, and citric acid^4^ as leaching agent. Recently review articles have summarized the findings of the studies targeting various approaches for the biomass valorization^19,30,31^. Briefly, Das et al.^25^ pre-treated sugarcane bagasse with deionized water, HCl, and HF and reported up to 46% ash removal with water (treated for 24 h), negative deashing with 5 M HCl, and 98.4% ash removal with 3% HF solution. A considerable mass loss due to hemicellulose and cellulose hydrolysis is also reported for acid leaching. Similarly, Chen et al.^32^ conducted hydrothermal carbonisation of sugarcane bagasse at a temperature of 180 °C for a duration of 30 min using a weak solution of sulfuric acid. They observed a decrease in the ash content from 3.55 wt% to 1.70 wt%. The treatment conditions including acid concentration, treatment time, and liquid to solid ratio widely vary between these studies. The resultant ash content thus varies too. It is worth noting that water washing may not be enough to reduce ash content to a level suitable for thermochemical technologies. On the other hand, an increased ash reduction along with a rapid treatment rate in case of acid treatment may be able to compensate for the higher treatment cost. The optimization of the process conditions can further reduce the treatment cost, which has rarely been addressed in the previous studies. Therefore, the exploration of demineralization technique using acidic reagents holds immense potential in terms of process optimization and cost-effectiveness. By reducing the mineral content in sugarcane bagasse, this process can unlock its potential as a sustainable and clean source of energy.

In our previous experiments, it was observed that leaching wheat straw, cotton straw, corncob, and coconut husk with dilute HCl solution for two hours at ambient temperature can significantly boost the heating values by reducing the ash content by more than 90%^2,33,34^. Despite an ash reduction of almost 70% with NaOH solution, these experiments further demonstrated the negative consequences of the basic treatment. Similar investigations are needed to establish treatment plans for additional biomass feedstocks that are widely accessible, like sugarcane bagasse and rice straw. Considering a notable gap in the literature, this study is aimed at investigating the valorization of sugarcane bagasse through acid leaching and identify the optimal operational conditions that result in the maximum reduction of ash content. The effect of operational variables including reagent concentration, reagent to solid ratio, and treatment time have been investigated and optimized using response surface methodology (RSM). Thermochemical and fusion characteristics of raw and sample treated at optimum conditions are thoroughly analyzed using techniques such as thermogravimetric analysis (TGA), Fourier-transform infrared spectroscopy (FTIR), ash fusion testing, proximate analysis, and calorimetry. The acquired results are anticipated to offer useful insights into the formulation of feedstocks for biomass-fired power plants.

## Materials and methods

### Sample collection and preparation

Sugarcane bagasse samples were gathered from local sugarcane juice vendors in Lahore, Pakistan. Prior to drying for 48 h at 45 °C, the samples were washed with distilled water to remove any surface dust particles. It is conceivable that this washing will not have a significant impact on either the mineral content or the elemental composition of the biomass. This is because the as-received sugarcane bagasse has a smaller surface area than the powdered samples, and the rinsing was done under normal environmental conditions. The biomass was first ground into a coarser powder, and then it was sieved through standard grit with a mesh number of 5, which produced particles of uniform size that were retained at the grit. Separately, dilute HCl solutions of desired concentration were prepared using a 37% HCl reagent obtained from Riedel–Dehaen.

### Design of experiments and statistical analysis

The response surface methodology (RSM) was used in this study to ascertain the conditions under which demineralization should be conducted most effectively. Experiment design used a Box-Behnken design (BBD) model with three factors and three levels. The independent parameters include treatment time (A), HCl molar concentration (B), and solid-to-liquid ratio (C). Ash removal percentage was used as a response variable and optimization objective. The lowest, the middle, and the highest levels of each factor were represented by the notations − 1, 0, and + 1 respectively. The corresponding parameter’s actual values are shown in Table 1. The range of these parameters is based on literature review and our prior experience of biomass demineralization^2,33–35^. The model suggested a total of 17 experiments. To determine the degree of correlation between the response and the process parameters, a polynomial equation was used as follows:

1 $$Y={\beta }_{0}+{\sum }_{i=1}^{N}{\beta }_{i}{X}_{i}+{\sum }_{i=1}^{N}{\beta }_{ii}{{X}_{i}}^{2}+{\sum }_{i=1}^{N-1}{\sum }_{j=i+1}^{N}{\beta }_{ij}{X}_{i}{X}_{j}$$

where *Y* is the expected response, *N* is the number of variables, *X*_*i*_ and *X*_*j*_ are the independent variables, ${\beta }_{0}$ is the intercept, ${\beta }_{i}$ and ${\beta }_{ii}$ denote the regression coefficients, and ${\beta }_{ij}$ is the interaction coefficient of independent parameters*.* The second-order model's fitness was statistically analysed using the analysis of variance (ANOVA) technique.

**Table 1:** Experimental levels of the independent variables.

Factor	Name	Unit	Low (− 1) Actual value	Center (0) Actual value	High (+ 1) Actual value
A	Treatment Time (min)	min	60	120	180
B	Concentration (M)	M	0.2	0.6	1
C	Solid: Liquid ratio	–	30	50	70

### Demineralization experiments

At room temperature and following the design of the experiment, a sample of 12 g of preconditioned sugarcane bagasse was added into a flask containing 250 ml of leaching reagents. The flask was positioned on an orbital shaker (Thomas-Scientific 1220A76) and subjected to a rotational speed of 250 revolutions per minute for a duration of 2 h in order to accelerate the leaching process. Following the leaching process, the solution underwent filtration to eliminate any surplus leaching agent. The solid residue was then washed with distilled water until it reached a neutral pH, effectively removing any remaining leaching reagent. Subsequently, the remaining substance was subjected to a 24-h drying process at a temperature of 105 °C in the oven, after which it was placed in a desiccator for further testing.

### Methods of characterizations

Loss-on-ignition analysis of oven-dried samples conducted in an air-muffle at 575 °C for 3 h was used to determine the ash content in raw and treated samples following ASTM E1755 (2007). The pre-dried sample was burned in the muffle furnace for 3 h at 575 °C, and the amount of residue (ash) was measured. Following the recommended protocol, it took a total of 5 h to get the results that indicated a difference of less than 0.3 mg between each successive reading. The values reported in this study are the average of triplicate tests. The definition of the percent ash reduction is then as follows:

2 $$Ash \,reduction \left(\%\right) = \left(1-\frac{demineralized\, biomass \,ash\, content}{raw\, biomass\, ash\, content}\right) 100$$

The selected sample, resulting in optimum ash reduction, was then used for additional testing and characterization. The bomb calorimeter (LECO; AC500 isoperibol calorimeter, Germany) was used to determine the biomass samples' higher heating value (HHV). The FTIR spectrometer (FTIR-Cary 630) was used to investigate the changes in the chemical structure of the biomass as a result of the inorganic leaching process.

The thermal degradation characteristics of the biomass samples were examined using thermogravimetric and differential thermogravimetric analysis (TGA and DTGA, respectively) using the SDT Q600 analyzer developed by TA instruments. A total of 10 mg of each sample was placed in a platinum crucible and subjected to heating at a temperature of 500 degrees Celsius in a nitrogen environment. Biomass was completely combusted by burning it in an oxygen-rich environment, with temperatures ranging from 500 to 950 °C, and a heating rate of 10 °C per minute. The equipment acquired the thermogram across the whole temperature range investigated in the experiment. In order to measure the rate of thermal degradation of both the untreated and treated samples, the data from TGA experiments were analysed using linear degradation models that have been previously documented^36^.

The ash fusion analysis was conducted with an ash fusion determinator (5E-AF4000; CKIC, China) in a strongly oxidizing environment. Following preparation in the air-muffle at 575 °C, the ash was finely ground until the particle size was reduced to less than 0.2 mm. The ash samples were blended with the binder solution, prepared by mixing 10 ml of deionized water and 1 g of dextrin, to make a thick paste. Before being introduced into the chamber of the ash fusion analyzer, the paste was first compressed using a cone-shaped mold with a height of 20 mm and a bottom measuring 6 mm. A high-resolution camera, integrated with the machine interface, captured the images of ash cones at every 10 °C temperature rise. The furnace was heated at a rate of 20 °C per min from 100 °C to 800 °C and 5 °C per min from 800 °C to 1500 °C. Deformation (DT), spherical (ST), hemispherical (HT), and flow temperatures (FT) were measured and analyzed.

### Statement regarding data collection

Permissions and guidelines were obtained from competent authority before collection of data and conduction of research.

## Results and discussion

The ash content of the raw sugarcane bagasse was found to be 4.16% on dry basis, which is in the range of reported values in the literature. The percent ash reduction of all 17 experiments, designed by the BBD method for the leaching process with different combinations of independent variables, is provided in Table 2. Every experiment was repeated at least three times, and average values are reported in the table. It can be observed that the ash reduction ranges between 57.83% to 83.87%. The maximum ash reduction is obtained when the biomass sample is treated for 120 min with 1 molar concentration of HCl and using a solid-to-liquid ratio of 30.

**Table 2:** Experimental Design and Results of Responses Variables.

Run	A (Time, min)	B (Concentration, M)	C (S/L ratio, wt/vol)	Y (% ash reduction)
1	60	0.6	70	64.29
2	60	1	50	78.03
3	120	1	70	76.37
4	180	0.6	70	71.11
5	120	0.2	30	72.64
6	120	0.6	50	71.42
7	120	0.6	50	70.19
8	120	0.6	50	71.17
9	180	0.6	30	75.16
10	60	0.2	50	58.68
11	120	1	30	83.87
12	180	1	50	79.94
13	120	0.6	50	71.03
14	120	0.2	70	57.83
15	180	0.2	50	70.76
16	60	0.6	30	75.52
17	120	0.6	50	72.99

### Fit summary

Table 3 presents a fit summary for the ash removal. The highest order polynomial model that is not aliased and terms are significant is chosen based on the Sequential Model Sum of Squares. As can be seen, the 2FI model is the most appropriate one to use when attempting to express ash reduction. The correlation between observed experiment results and model-predicted values can be evaluated using the determination coefficient (R^2^). The high R^2^ values and small differences between predicted and adjusted R^2^ indicate a strong correlation between the experimental data and the model's predicted values, indicating that the model is well-fitted. The R^2^, Adjusted R^2^, and Predicted R^2^ for the 2FI model in this study are found to be 0.97, 0.95, and 0.87, respectively. Whether or not additional or higher-order terms are statistically significant, doing so will always improve the R^2^ value of the model. High values for Adjusted R^2^ and Predicted R^2^ are thus used to demonstrate the model's acceptability.

**Table 3:** Multi-regression analysis of the objective function (% ash reduction).

Source	Sum of Squares	Degree of freedom	Mean square	F-value	p-value	Comments
Sequential model sum of squares						
Mean vs. Total	87,696.53	1	87,696.53			
Linear vs. Mean	653.76	3	217.92	38.23	< 0.0001	
** 2FI vs. Linear**	**52.1**	**3**	**17.37**	**7.9**	**0.0054**	**Suggested**
Quadratic vs 2FI	4.36	3	1.45	0.5774	0.6481	
Cubic vs. Quadratic	13.46	3	4.49	4.3	0.0964	Aliased
Residual	4.17	4	1.04			
Total	88,424.39	17	5201.43			
Lack of fit tests						
Linear	69.93	9	7.77	7.44	0.0344	
** 2FI**	**17.82**	**6**	**2.97**	**2.85**	**0.1653**	**Suggested**
Quadratic	13.46	3	4.49	4.3	0.0964	
Cubic	0	0				Aliased
Pure error	4.17	4	1.04			

Model summary statistics						
Source	Std. Dev	R^2^	Adjusted R^2^	Predicted R^2^	PRESS	Comments
Linear	2.39	0.8982	0.8747	0.7939	150.05	
**2FI**	**1.48**	**0.9698**	**0.9517**	**0.8737**	**91.96**	**Suggested**
Quadratic	1.59	0.9758	0.9446	0.6952	221.84	
Cubic	1.02	0.9943	0.9771		*	Aliased

Significant values are in bold.

### Analysis of variance and model fitting

Analysis of variance (ANOVA) was used to determine whether the coefficients of the models are statistically significant, and results are shown in Table 4 for the 2FI model. The P-value, which signifies the significance of the coefficients, is essential for understanding the relationship between independent variables^37^. The 2FI model is determined to be statistically significant, as indicated by its P-value being less than 0.0001. The F-value of 38.23 also suggests that the 2FI model is significant. The likelihood of an F-value of this size occurring due to random noise is only 0.01%. In addition, the Lack of Fit F-value of 7.44 indicates that the Lack of Fit is not statistically significant compared to the pure error. The reliability of the experiment is indicated by having a lower value of the coefficient of variation (CV). A low CV value (2.06%) found in this study confirms the reproducibility of the results. Low PRESS values further imply the usefulness of the models for predictive applications. The statistical values of the 2FI model parameters are also shown in Table 4. All the model terms, both linear and non-linear, can be seen to significantly affect the response.

**Table 4:** Results of ANOVA for the 2FI model.

ANOVA of the 2FI model						
Source	Sum of squares	Degree of freedom	Mean Square	F-value	p-value	Comments
Model	705.87	6	117.64	53.49	< 0.0001	Significant
A: Treatment time	52.28	1	52.28	23.77	0.0006	
B: Concentration	424.86	1	424.86	193.16	< 0.0001	
C: Solid-to-liquid ratio	176.63	1	176.63	80.3	< 0.0001	
AB	25.86	1	25.86	11.76	0.0065	
AC	12.89	1	12.89	5.86	0.036	
BC	13.36	1	13.36	6.07	0.0334	
**Residual**	22	10	2.2			
Lack of Fit	17.82	6	2.97	2.85	0.1653	Not significant
Pure Error	4.17	4	1.04			

**Figure 1:**
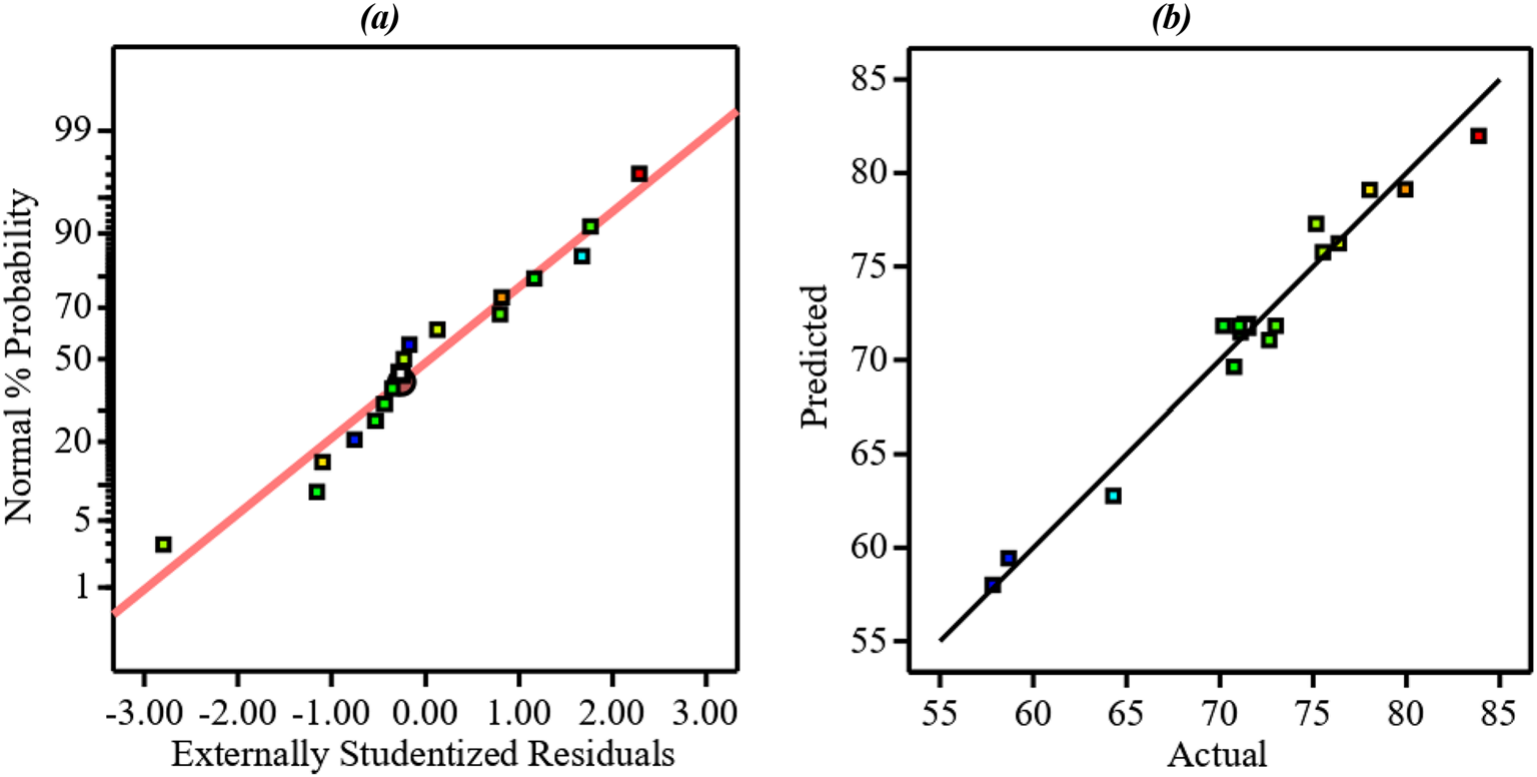
Diagnostic test of the model fitting. (**a**) Normal probability plot of the internally studentized residuals and (**b**) Predicted vs. Actual response.

The adequacy of the suggested model is also assessed by the normal probability plot of the internally studentized residual and predicted vs. actual response, as shown in Fig. 1. As can be observed, the linear trend in the probability plot and close correspondence between predicted vs. actual response confirm the adequacy of the 2FI model.

Besides the 2FI model, regression was also done with the quadratic model to assess whether adding additional non-linear terms has any statistically significant effect on the prediction of percent ash reduction. The results, not shown here, revealed that none of the additional quadratic terms have a P-value less than 0.05 suggesting an insignificant contribution. Subsequently, the 2FI model is selected for further analysis. The final empirical model, obtained after optimization and model validation, in terms of actual factors and units, as mentioned in Table 2, is given below:

3 $$Y=75.7272+0.0313\text{A}+19.5093\text{B}-0.5515\text{C}-0.1059\text{AB}+0.0015\text{AC}+0.2284\text{BC}$$

### Response surfaces

**Figure 2:**
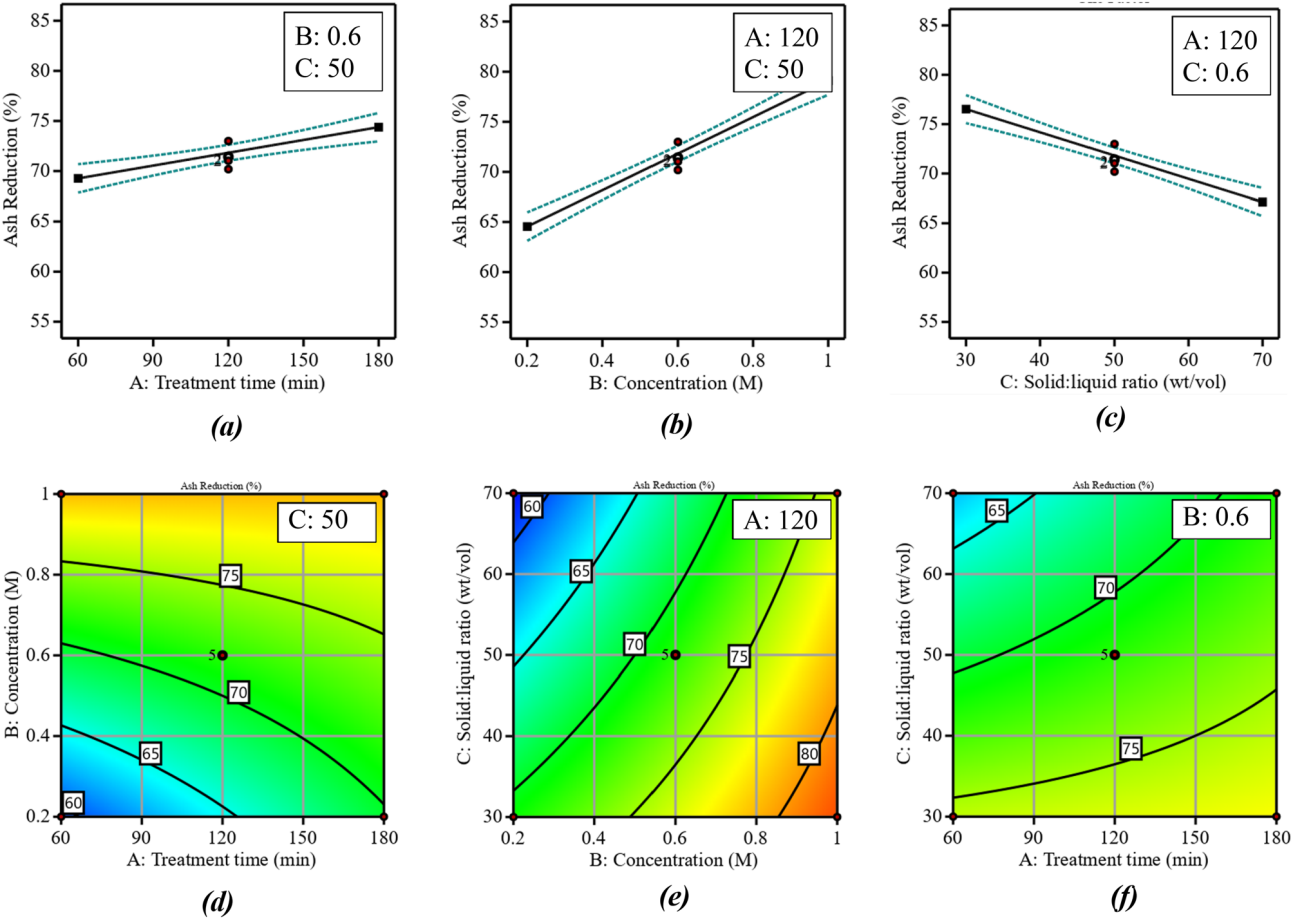
Single factor effect (**a**–**c**) and combined factor effect (**c**–e) on the percent ash reduction.

The developed model was further used to investigate the individual and interaction effects of the independent parameters on the ash reduction; the results are shown in Fig. 2. The axis values are the real values, and the constant parameter values are provided as an insert in each graph. It can be observed from Fig. 2a–c that all variable parameters, including reagent concentration, treatment time, and the solid-to-liquid ratio, have a considerable effect on the ash reduction. Increasing both reagent concentration and treatment time increases ash reduction. The solid-to-liquid ratio, however, inversely affects the ash reduction as a higher solid-to-liquid ratio makes it more difficult to mix the sample with the reagent, which tends to reduce the likelihood that any soluble inorganics will be extracted. The effect of concentration is among the highest, whereas the treatment duration is of the least influence. This relationship is also statistically supported since the reagent concentration has the highest F-value (193.16), followed by treatment time (80.3) and solid-to-liquid ratio (23.77). The iso-response of the combined effect is shown in Fig. 2d–f. The F-value of AB, BC, and AC factors are 11.76, 6.86, and 5.86, respectively, suggesting the significance and strength of the combined effect of interaction parameters. For instance, the ash reduction monotonously increases by increasing the concentration and decreasing the solid-to-liquid ratio, as evident from the diagonal iso-response lines of Fig. 2e.

### Optimization by response surface modeling

Using a statistical method, ANOVA, independent variables including treatment time, concentration, and solid-to-liquid ratio have been optimized for maximum ash reduction. The model suggested at least 10 experimental designs leading to optimum conditions, and the set with minimum treatment time was selected, as shown in Table 5, along with the experimental response. The selection of the minimum treatment time (80 min) among the optimal designs reflects a trade-off between maximizing ash reduction and minimizing processing time. While longer durations might lead to slightly higher demineralization levels, the economic implications of extended processing times (e.g., energy consumption) were considered. The predicted and experimental results are also reported in the table. The predicted value of ash reduction at optimum conditions was 79.1%. The acid leaching experiment was then performed at predicted recommended conditions for the validation purposes. The experimental ash reduction found at optimum conditions was 78.2% thus giving a very close correspondence between predicted and measured ash reduction. The comparison between the experimental and predicted findings reveals a discrepancy of 0.01%. Based on the results, it can be inferred that the model developed has the ability to reliably forecast the ash reduction. It is important to acknowledge that this study was conducted in a controlled laboratory setting. The applicability of these findings for large-scale industrial implementation necessitates further research.

**Table 5:** Optimal demineralization conditions and model validation.

Treatment time (min)	Concentration (M)	Solid-to-liquid ratio (wt/vol)	% Ash reduction	
			Predicted mean	Experimental mean
80	1	50	79.1	78.2

### Characterization of biomass valorization

#### Effect of demineralization on the lignocellulosic composition and heating value

Demineralization leaches out the mineral contents, increasing the biomass's organic fraction. Consequently, higher heating value (HHV) is increased, and slagging nature of the biomass and surface corrosion caused by high-temperature thermochemical conversion is decreased. Besides leaching free and bonded alkali and alkaline earth metals, strong acids and bases can potentially hydrolyze the organic structure, thus lowering the mass and energy yield. The lignocellulosic composition and HHV of raw and demineralized biomass samples are shown in Table 6. The dry content of raw samples was 93.3%. The chemical composition of sugarcane bagasse was analyzed for cellulose, hemicellulose, lignin, and ash. The most abundant component was cellulose, which made up 40.34% of the total, followed by hemicellulose, 38.07%, and lignin (21.59%). The percentage of sugarcane bagasse's total solid content comprised of cellulose and hemicelluloses was 78.41%. For efficient valorization, selecting a suitable leaching agent is essential, as the use of an incorrect reagent may result in the dissolution of lignin, cellulose, and hemicellulose, hence decreasing the value of the biomass. The composition of the lignocellulosic material is significantly altered due to the acid pre-treatment. As a likely consequence of organic compounds being dissolved in an acid medium, the hemicellulose fraction dropped from 38.07% to 32.87%. However, the HHV value of treated demineralized sugarcane bagasse increases by 9.82% (from 16.2 MJ/kg to 17.8 MJ/kg). Das et al.^25^ previously reported a 10% drop in the heating value of sugarcane bagasse during demineralization with 5 M HCl solution for 1 h. The heating value drop was associated with severe hydrolysis of hemicellulose structures. Conversely, Asadieraghi et al.^38^ reported that the acid treatment not only increases the heating value but also results in better thermal stability of the biomass, which is necessary for controlled combustion in the boiler.

**Table 6:** Chemical composition and higher heating value of raw and demineralized sugarcane bagasse samples.

Biomass	Lignocellulosic chemical composition			HHV (MJ/kg)
	Hemicellulos (wt %)	Cellulose (wt %)	Lignin (wt %)	
Raw sample	38.07	40.34	21.59	16.2
Demineralized sample	32.87	45.04	22.09	17.8

#### Effect of demineralization on ash fusibility

**Figure 3:**
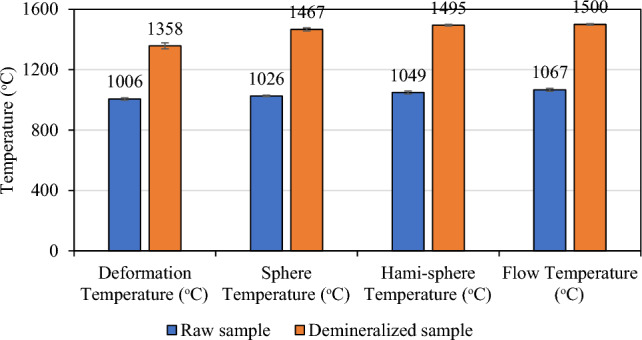
Ash fusion temperatures of raw and demineralized biomass samples.

Ash fusibility is an important factor for predicting ash behavior during thermochemical conversion. The measurements of the ash fusion temperature are carried out with a maximum temperature of 1500 °C in an oxidizing environment. During the testing process, the deformation, softening, hemispheric, and fluid temperatures are measured and recorded according to the cone shapes placed on the sample holder. The ash fusibility results are shown in Fig. 3. It can be observed that the ash deformation of the raw sample starts at 1006 °C, and the fluid temperature approaches 1067 °C. The combustion temperature of small-scale household boilers is typically 1000 to 1200 °C, suggesting that sugarcane bagasse may result in a significant amount of fouling and slagging in such boilers. Jenkins et al.^39^ and Wu et al.^12^ reported that removing ash content via leaching evidently improves the melting temperature. Consistently, the deformation temperature increased from 1006 °C to 1358 °C, and similarly, the fusion temperature increased from 1067 °C to higher than the test limits (1500 °C). Ash melting is unlikely to take place during combustion because fusion temperatures of the demineralized biomass are significantly higher than the combustion temperatures of small-scale household boilers. This will prevent boiler issues related to ash, such as slagging and agglomeration.

#### Effect of demineralization on chemical structure

**Figure 4:**
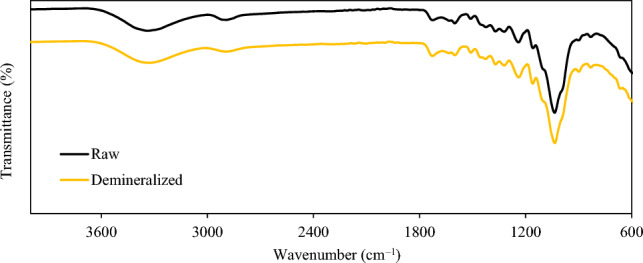
FTIR spectra of raw and selected demineralized samples.

Infrared spectroscopy is commonly used to investigate the chemical structure of lignocellulosic biomass samples. In this study, FTIR was used to evaluate the impacts of ash removal on the sugarcane bagasse chemical structure. The resultant FTIR spectra of raw and demineralized samples are shown in Fig. 4. The first band in FTIR spectra is found between 3600 and 3000 cm^−1^ and is attributable to the O–H bond stretching of the –OH functional group (alcoholic, carboxylic, or phenolic structures). The second band is between 3000 and 2800 cm^−1^ and is assigned to the C-H bond stretching vibration of –CH_2_ and –CH_3_ functional groups. Following demineralization, both bands showed a modest reduction, which is consistent with the findings of Asadieraghi et al.^38^ and Fierro et al.^40^. The third band can be seen between 1700 and 1800 cm^−1^ and is associated with the C=O bond stretching of carboxyl groups. This band is typically present in the structure of hemicellulose. Previously, it was observed that the basic treatment has a far more profound impact on the hemicellulose component than the acidic treatment^34^. The spectra appearing below 1400 cm^−1^ are typically associated to amide or sulfamide bond, C–O stretching of COOH, and –CN stretching. Besides small alterations in the intensity of band peaks, no significant or noticeable effect of acid leaching has been observed in the chemical structures. The slight decrease in the intensity of higher wavenumber peaks is expected to arise from the primarily hemicellulose fraction of the biomass. This is consistent with the chemical composition observation reported in Table 6.

#### Effect of demineralization on the thermal degradation behavior

**Figure 5:**
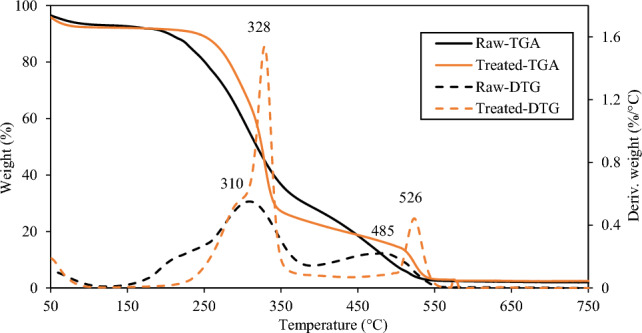
TGA and DTGA curves of raw and treated samples.

Demineralization's impact on the thermal degradation behavior of sugarcane bagasse was investigated using TGA. The curves for mass loss (TGA) and derivative mass loss (DTGA) for untreated and treated samples are shown in Fig. 5. Thermogram can be divided into four regions: region-I, below 150 °C, is caused by the removal of moisture and light hydrocarbons; region-II, between 150 and 400 °C, is attributed to the degradation of hemicellulose and cellulose, region-III, from 400 to 600 °C, is caused by the slow degradation of lignin content, and region-IV, above 600 °C, represents the remaining non-combustible fraction of the biomass shown by flat tailing section. The region-II and region-III are related to the combustion process, making the corresponding peak of particular interest in the demineralization and thermochemical applications of biomass. When the organic volatiles are released in the region-II, a high-intensity peak in the DTG profile is observed containing a lower temperature shoulder. The shoulder represents the decomposition of hemicellulose, while the main peak is associated with cellulose decomposition. The shoulder of the treated sample has skewed, supporting the observation made in Section "Effect of demineralization on the lignocellulosic composition and heating value" that a fraction of the hemicellulose has been hydrolyzed during the leaching process. Further, it can be observed that the peaks corresponding to both combustible regions have shifted to the higher temperature for the treated sample compared to the raw sample, i.e., the peak temperatures of cellulose degradation shifted by 18 °C and the peak temperatures of lignin degradation shifted by 41 °C. During thermochemical conversion, some of the minerals present in the biomass act as a catalyst, and upon their removal, the biomass becomes more resistant to thermochemical decomposition, which is reflected by increased peak temperature to a higher level^41^.

The rate of thermal degradation ($d\alpha /dt$) of biomass can be expressed by the following expression:

$$\frac{d\alpha }{dt}=\text{Aexp }\left(-\frac{E}{RT}\right).f\left(\alpha \right),$$

where A is the frequency factor and $\alpha =\left({w}_{0}-w\right)/ \left({w}_{0}-{w}_{f}\right)$ is the residual mass fraction determined from the initial (${w}_{0}$), final ${w}_{f}$, and instantaneous ($w$) mass of the sample.

For non-isothermal TGA study following constant heating rate (*β*), ($d\alpha /dT$) can be expressed as;

$$\frac{d\alpha }{dT}=\frac{\text{A}}{\beta }\text{exp }\left(-\frac{E}{RT}\right).g\left(\alpha \right).$$

The g(α) is the mechanism function and can be implicated by different reaction models and expressions. The integration produces a linear equation $ln\left(g(\alpha )/{T}^{2}\right)=ln\left(AR/\beta E\right)\left(1-2RT/E\right)- E/RT$. The activation energy (*E*) can be determined from the slope between $ln\left(g(\alpha )/{T}^{2}\right)$ vs $1/T$ and pre-exponent factor (*A*) can be determined from the reduced form of intercept $ln\left(AR/\beta E\right)$ assuming 2*RT* <  < *E*. Since this stage is a more active mass-loss region in thermochemical conversion, the kinetic parameters have been determined for region-II (150–400 °C). In this study, five different reaction models, listed in in Table 7, have been adopted from the literature^36^ to determine the kinetic parameters of thermal degradation. The appropriateness of the mechanism function model can be examined from the linearity of *ln[g(α)/T2]* versus *1/T* and the value of the coefficient of determination (R^2^). The kinetic parameters obtained by fitting models to the TGA data of raw and demineralized samples are shown in Table 7. The R^2^ values for raw samples are in the range of 0.96–0.97 and for treated samples the value is 0.93–0.94 which suggests the appropriateness of the considered models. The activation energy of raw and treated samples is in the range of 44.53–61.14 kJ/mol and 66.28–95.66 kJ/mol, respectively. Clearly, the activation energy of treated samples increased by more than 20 kJ/mol due to demineralization, which supports the argument of increased thermal stability due to the removal of minerals. Similarly, the rate constant of the treated sample has dropped to almost two orders of magnitude, indicating a delayed and slow degradation rate of demineralized samples compared to the raw sample. Since minerals in biomass act as catalysts during thermal degradation, their removal inhibits and slows down the degradation.

**Table 7:** Kinetic parameters of raw and demineralized samples determined from the Coats-Redfern model based on thermogravimetric data of region-II (150–400 °C).

Model	g(α)	Raw sample			Demineralized sample		
		E (kJ/mol)	k (min^−1^)	R^2^	E (kJ/mol)	k (min^−1^)	R^2^
Parabolic rule	α^2^	44.53	0.77	0.97	66.28	8.05 × 10^−3^	0.94
Va lensi equation	α + (1 − α)ln(1 − α)	48.12	0.55	0.97	72.37	3.39 × 10^−3^	0.94
Ginstling-Broushtein equation	(1–2/3α) − (1 − α)^2/3^	49.49	1.68	0.97	74.78	8.28 × 10^−3^	0.94
Jander equation	[1 − (1 − α)^1/3^]^2^	52.30	0.77	0.97	79.74	2.41 × 10^−3^	0.93
ZLT equation	[1/(1 − α)]^1/3^–1]^2^	61.14	0.06	0.96	95.66	6.79 × 10^−3^	0.93

## Conclusion

Sugarcane bagasse samples were demineralized with inexpensive and commercially available dilute solutions of HCl. The response surface methodology following the Box Behnken Design (BBD) model and analysis of variance (ANOVA) were used for experiment design, statistical analysis, and determination of the optimum conditions. It has been found that the reagent concentration and treatment time has direct effect on the ash reduction whereas solid-to-liquid ration inversely affects the ash reduction. The optimum conditions for maximum ash reduction (78.2%) were found to be 1 M HCl concentration, 80 min treatment time, and a solid-to-liquid ratio of 50. The effect of acid leaching on the chemical, thermal, and fusion characteristics was also evaluated. The hemicellulose content of the treated sample slightly decreased compared to the lignocellulosic composition of the raw biomass. It is expected that a fraction of hemicellulose is hydrolyzed in the presence of acidic media. The effect was also observed the FTIR spectra. The higher heating value, however, increased by 9.82% which is primarily ascribed to the removal of minerals and low heating value organic content. The combustion temperatures of the treated samples, including deformation, softening, hemispheric, and fluid temperatures, significantly increased compared to the raw sample suggesting thermal stability. The same effect has been observed in the TGA analysis as the peak temperatures of the thermal degradation of demineralized biomass considerably increased. The thermogram of raw and treated samples were also fitted to linear thermokinetic models to quantify the kinetic parameters. It was found that the activation energy of the major peak of the treated sample, corresponding to the degradation of hemicellulose and cellulose, increased by more than 20 kJ/mol, and the pre-exponent factor decreased by almost two orders of magnitude. These values strongly support the argument of enhanced thermal stability of the treated sample caused by the removal of the alkali and alkaline earth metals.

The present study holds significant importance in the field of sugarcane bagasse demineralization, particularly in the context of Pakistan's cane sugar production and consumption. However, the findings and optimization parameters derived from this study can also be extrapolated and applied to similar biomass feedstocks and thermochemical conversion processes in other regions globally, providing valuable insights. While this study has yielded valuable findings, it is crucial to acknowledge its limitations and address them for the commercial application of the proposed solution. Firstly, the study was conducted under controlled laboratory conditions, and the scalability and practical implementation of the proposed approach on an industrial scale were not assessed. Therefore, further research is required to evaluate power consumption for stirring and post-treatment of the spent leaching agent to determine the commercial viability. Moreover, the study primarily focused on the effects of treatment time, solid-to-liquid ratio, and acid concentration at ambient conditions, while possible effect of temperature was not considered. Future work should consider these factors to comprehensively assess the economic viability of the proposed approach.

## Data Availability

Data will be made available upon reasonable request from the corresponding author.
